# Sentence-Level Silent Speech Recognition Using a Wearable EMG/EEG Sensor System with AI-Driven Sensor Fusion and Language Model

**DOI:** 10.3390/s25196168

**Published:** 2025-10-05

**Authors:** Nicholas Satterlee, Xiaowei Zuo, Kee Moon, Sung Q. Lee, Matthew Peterson, John S. Kang

**Affiliations:** Department of Mechanical Engineering, San Diego State University, San Diego, CA 92182, USA; nsatterlee0532@sdsu.edu (N.S.); xzuo1800@sdsu.edu (X.Z.); kmoon@sdsu.edu (K.M.); sqlee@sdsu.edu (S.Q.L.); mpeterson0509@sdsu.edu (M.P.)

**Keywords:** silent speech recognition, EEG, EMG, few-shot learning, sensor fusion, language model

## Abstract

Silent speech recognition (SSR) enables communication without vocalization by interpreting biosignals such as electromyography (EMG) and electroencephalography (EEG). Most existing SSR systems rely on high-density, non-wearable sensors and focus primarily on isolated word recognition, limiting their practical usability. This study presents a wearable SSR system capable of accurate sentence-level recognition using single-channel EMG and EEG sensors with real-time wireless transmission. A moving window-based few-shot learning model, implemented with a Siamese neural network, segments and classifies words from continuous biosignals without requiring pauses or manual segmentation between word signals. A novel sensor fusion model integrates both EMG and EEG modalities, enhancing classification accuracy. To further improve sentence-level recognition, a statistical language model (LM) is applied as post-processing to correct syntactic and lexical errors. The system was evaluated on a dataset of four military command sentences containing ten unique words, achieving 95.25% sentence-level recognition accuracy. These results demonstrate the feasibility of sentence-level SSR using wearable sensors through a window-based few-shot learning model, sensor fusion, and ML applied to limited simultaneous EMG and EEG signals.

## 1. Introduction

Silent Speech Recognition (SSR) is an emerging technology that enables the interpretation of speech content without the need for audible vocalization. Unlike traditional Automatic Speech Recognition (ASR) systems, which depend on acoustic signals to transcribe spoken language, SSR systems leverage non-acoustic biosignals generated during speech production. This distinction makes SSR particularly advantageous in scenarios where vocalization is impractical or undesirable, such as high-noise environments, situations requiring confidentiality, or communication involving individuals with speech impairments. Consequently, SSR has the potential to support a wide range of applications in the medical, military, and industrial sectors.

Current SSR systems predominantly utilize two biosignal modalities: electromyography (EMG), which captures articulatory muscle activity, and electroencephalography (EEG), which records neural activity related to speech planning and execution. Among these, EMG-based SSR has demonstrated particularly strong performance in isolated word recognition tasks.

Early work by Sugie et al. [1] demonstrated an EMG-based system that classified five Japanese vowels with 64% accuracy. Cha et al. [2] employed multichannel facial surface EMG (sEMG) combined with a bidirectional long short-term memory (Bi-LSTM) network to classify six Korean command words, achieving 92.5% accuracy. Zhu et al. [3] used high-density sEMG along with linear discriminant analysis to classify English digits from “zero” to “nine”, reporting accuracies between 87% and 90%. Similarly, Deng et al. [4] applied convolutional neural networks (CNNs) to identify 33 Chinese words with an accuracy of 88%. Schultz et al. [5] developed an EMG-based SSR system capable of achieving a word error rate (WER) as low as 10% for the best-performing speaker on a 101-word English vocabulary task. Sonawane et al. [6] created a two-channel EMG system utilizing an artificial neural network to classify four labial words in Devanagari, a North Indian script. Maier-Hein et al. [7] employed a hidden Markov model (HMM) with seven sEMG channels to classify English digits from “zero” to “nine”, achieving an accuracy of 97.3%. Jorgensen et al. [8] demonstrated an EMG-based neural network system that classified six English command words (“stop”, “go”, “left”, “right”, “alpha”, and “omega”) with an accuracy of up to 92%.

EEG-based SSR approaches have also demonstrated promising results, particularly in classifying isolated words from non-vocal neural activity. These methods leverage EEG’s ability to capture brain signals associated with speech planning and internal articulation, providing a complementary modality to EMG.

Porbadnik et al. [9] employed a 16-channel EEG setup combined with HMM to classify five English words (“alpha”, “bravo”, “charlie”, “delta”, and “echo”). González-Castañeda et al. [10] achieved 83.3% accuracy in classifying five Spanish words using EEG signals. Vorontsova et al. [11] integrated CNNs with recurrent neural networks to classify nine Russian words, reporting 85% accuracy. Duraisamy et al. [12] applied transfer learning with a Bi-LSTM architecture, attaining nearly 80% accuracy on five English command words (“left”, “right”, “up”, “pick”, and “push”). Balaji et al. [13] recognized two English and two Hindi words using a neural network model, reporting 75.4% accuracy. Singh et al. [14] focused on classifying one long versus one short English word, achieving a mean classification accuracy of 85%. Pawar et al. [15] analyzed EEG signals associated with directional words (“left”, “right”, “up”, and “down”), reaching 85.6% accuracy. Wang et al. [16] studied the classification of two Chinese characters, with individual subject accuracies ranging from 73.65% to 95.76%.

Transitioning to sentence-level SSR introduces substantial challenges, particularly in segmenting continuous biosignals into discrete word units. Coarticulation effects—where the articulation of one word influences the production of the next—can blur word boundaries in EMG and EEG recordings, making segmentation and classification considerably more difficult. While a number of studies have explored word recognition from sentence-level signals, the literature on full sentence recognition using wearable EMG or EEG sensors remains limited.

Early work by Suppes et al. [17] demonstrated the feasibility of sentence-level recognition using EEG signals. In their study, sixteen scalp-mounted EEG sensors were used in conjunction with optimal predictive filters to classify twelve distinct auditory sentences that included short pauses between words. Deng et al. [18] utilized a subspace Gaussian Mixture Model to classify common English phrases and text message sentences from four-channel sEMG signals, achieving an accuracy of 88.8%. Meltzner et al. [19] achieved 91.1% accuracy in classifying English phrases derived from a 2200-word vocabulary using an 11-channel sEMG sensor system. Their system recorded EMG signals from articulator muscles concurrently with acoustic signals to aid supervised learning. Alharbi et al. [20] developed a hybrid deep learning model that classified five English expressions (“hello”, “help me”, “stop”, “thank you”, and “yes”) with 77.8% accuracy.

While these studies report promising results, most rely on high-density, multi-channel sensor systems that are not readily wearable, or they achieve limited classification accuracy. These constraints pose challenges for deployment in practical, real-world contexts—particularly in secure, mobile, or military environments.

Sensor fusion offers a promising path to improve SSR accuracy and robustness, yet its application remains relatively underexplored. For instance, Li et al. [21] developed a hybrid SSR interface that combined sEMG and EEG signals, achieving 92.8% accuracy in Chinese character recognition. However, their system used a high-density configuration—57 EEG channels and 5 EMG channels—and required parallel acquisition of audible speech for training. These constraints limit its viability for wearable, unobtrusive, or silent use cases.

Language models (LMs) represent another powerful method for improving sentence-level SSR accuracy. In ASR, LMs are routinely employed to refine the output of acoustic models by leveraging syntactic and semantic structure. For example, Messaoud et al. [22] demonstrated a ~3% accuracy gain using a bigram model; Dutta et al. [23] reported consistent reductions in word error rate (WER) using pre-trained encoder–decoder models; and Salazar et al. [24] applied transformer-based architectures such as BERT to improve hypothesis selection. D’Haro et al. [25] used phrase-based machine translation for error correction, further showcasing the versatility of LMs in post-processing.

Because LMs operate independently of input modality, their benefits can naturally extend to SSR. Wadkins [26] showed that applying an n-gram LM to EMG-based SSR reduced the classification error by 4–15%. Similarly, Wand et al. [27] achieved 89.1% sentence-level accuracy on a 108-word English vocabulary by combining Principal Component Analysis and a trigram LM. Their system, however, required a 16-channel EMG array and audible speech recordings during training, limiting its adaptability. Despite their success in ASR, LMs remain significantly underutilized in SSR research.

SSR research also faces significant challenges related to data scarcity. High-quality biosignal datasets are difficult and time-consuming to collect, often resulting in small sample sizes and limited vocabulary coverage. These constraints pose a major obstacle to the use of conventional machine learning approaches, which typically require large volumes of labeled data to perform effectively.

To address these limitations, this study presents a novel SSR framework that combines sensor fusion, few-shot learning, and LM for wearable, sentence-level SSR. We propose a lightweight wearable sensor system that integrates a single-channel EMG and a single-channel EEG sensor, both equipped with real-time wireless data transmission capabilities. This configuration ensures practicality for real-world applications while maintaining a minimal hardware footprint.

Our model leverages a Siamese neural network (SNN)-based few-shot learning architecture, which is specifically designed to operate efficiently on limited data. The system integrates EMG and EEG inputs using a previously developed Parallel Multi-Layer Sensor Fusion (PMLSF) method, which leverages the complementary strengths of both modalities: EMG captures articulatory muscle activity, while EEG reflects underlying neural processes involved in speech planning.

A detailed description of the system architecture is presented in Section 2, covering the experimental setup, data collection procedures, data augmentation strategies, implementation of few-shot learning, moving window-based segmentation, the sensor fusion method, and the integration of the LM. Section 3 presents experimental results, evaluating model performance on both isolated word and sentence-level classification tasks. Section 4 offers a detailed discussion of the findings, and Section 5 concludes the study by summarizing key contributions and outlining directions for future work.

## 2. Methods

### 2.1. Wearable and Wireless EEG/EMG Sensor System

**Figure 1:**
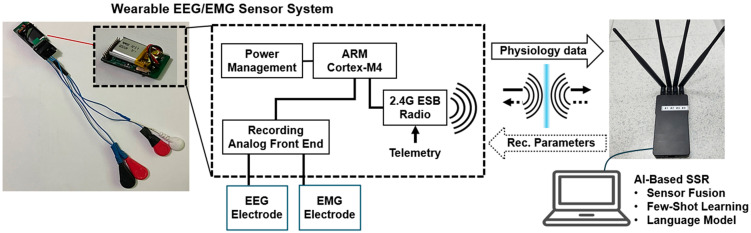
Schematic diagram of the wearable and wireless EEG/EMG sensor system.

For data acquisition, we employed a compact, wireless EEG/EMG sensor system featuring one channel each of EEG and EMG, as illustrated in Figure 1. Each sensor module included a bipolar electrode configuration, consisting of one signal electrode and one reference electrode. Both neural (EEG) and muscular (EMG) signals were captured using the RHD2216 chip from Intan Technologies (Los Angeles, CA, USA), a low-power, 16-channel differential amplifier with an integrated 16-bit analog-to-digital converter (ADC). The chip supports high-resolution acquisition at a sampling rate of 4 kHz per channel, making it suitable for both EEG and EMG signals, particularly in wearable systems with short inter-electrode distances. Its low-noise architecture and programmable amplifiers ensure high signal fidelity across modalities.

The system also integrates the nRF52832 System-on-Chip (SoC) from Nordia Semiconductor (Trondheim, Norway), which handles both processing and wireless communication. Custom firmware was developed to optimize the trade-off between computational performance and energy efficiency, enabling continuous operation in a low-power wearable form factor.

An interrupt service routine (ISR) runs at 4 kHz to synchronize data acquisition with the RHD2216 chip via the Serial Peripheral Interface (SPI). During each ISR cycle, the chip acquires biosignal data and can update filter configurations. Once the data buffer reaches a preset threshold, it is transmitted via Bluetooth Low Energy (BLE) to a host PC for real-time processing and classification.

A complete specification of the sensor hardware components is provided in Table 1.

**Figure 2:**
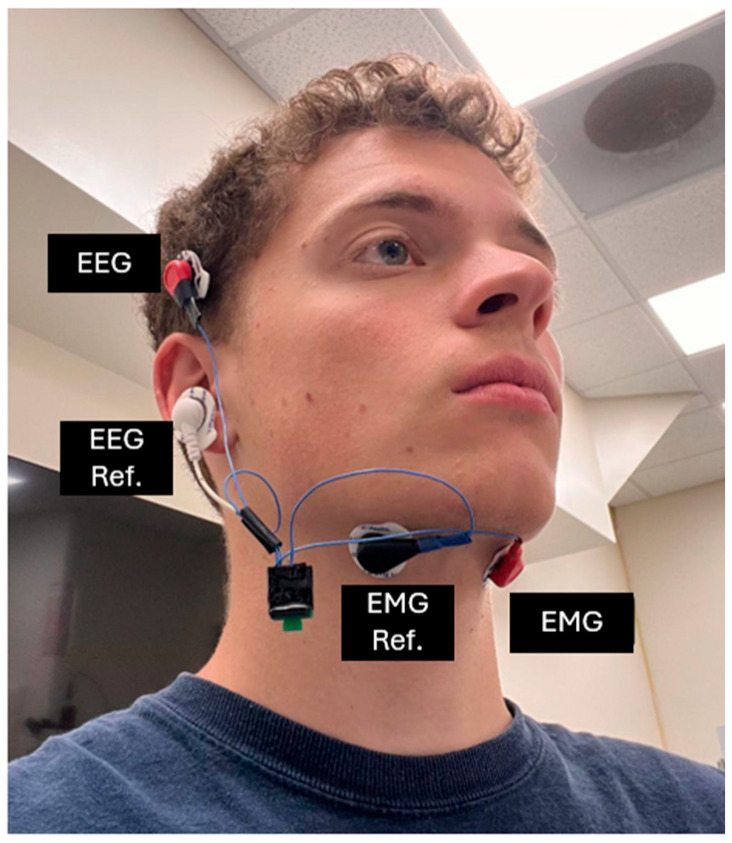
EEG/EMG sensor placement.

As illustrated in Figure 2, the EEG and EMG sensors were positioned based on both anatomical relevance and functional considerations. The EEG recording electrode was placed on the skin superior to the ear, targeting a region adjacent to the primary auditory cortex, located on the upper bank of the superior temporal gyrus within the superior temporal lobe. The reference electrode for EEG was placed on the lobule of the ear to ensure a stable baseline signal.

For EMG acquisition, electrodes were positioned bilaterally beneath the chin, targeting the extrinsic tongue muscles—specifically the genioglossus and hyoglossus. These muscles, originating from the mandible and hyoid bone, play a critical role in articulatory movement during both vocalized and silent speech. EMG signals were captured in differential mode to enhance signal quality and minimize noise artifacts.

### 2.2. Data Collection

Military operations often require silent communication or communication in noisy environments, where conventional vocal speech is either impractical or compromised. Moreover, such contexts demand highly accurate information exchange. To evaluate the effectiveness of silent sentence recognition, four representative command phrases were selected from the U.S. Army manual *Visual Signals* (TC 3-21.60): “Need Medical Assistance”, “Land Here”, “Do Not Land Here”, and “Pick Us Up”.

Using the EMG/EEG sensor system, two datasets were collected from a 21-year-old male participant. The first dataset (Corpus A) consists of EMG and EEG recordings of individual words extracted from the selected sentences: “Need”, “Medical”, “Assistance”, “Land”, “Here”, “Do”, “Not”, “Pick”, “Us”, and “Up”. The second dataset (Corpus B) comprises continuous EMG/EEG recordings of the four full sentences, enabling analysis of connected word sequences in natural speech.

**Figure 3:**
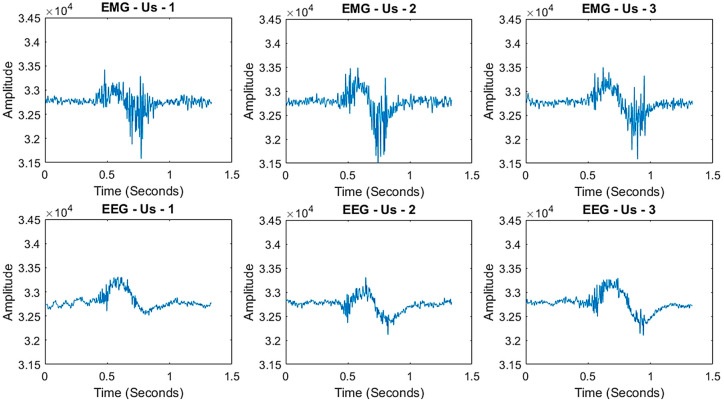
Individual word recordings of the words “Us”.

Corpus A consists of 1500 samples, with 150 recordings for each of the ten target words. This dataset is used to pretrain the SNN model for individual word recognition. Figure 3 illustrates example recordings of the word “Us”. While the overall signal patterns are consistent, slight variations are observed across different instances of the same word, reflecting natural variability in EMG and EEG signals.

**Figure 4:**
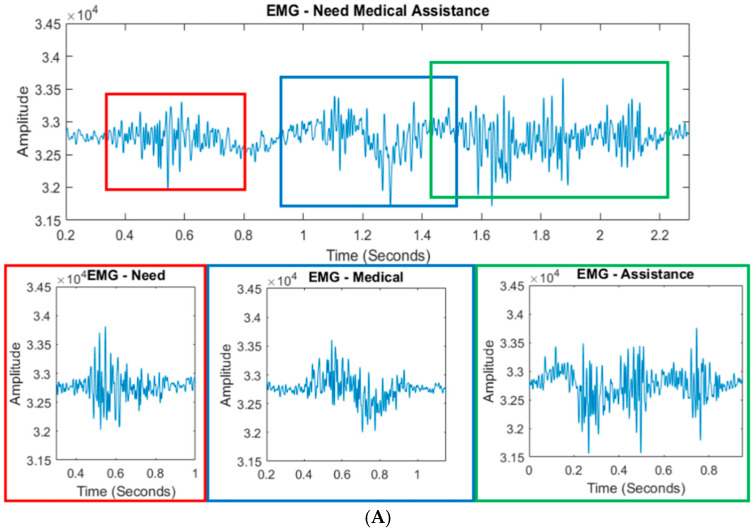

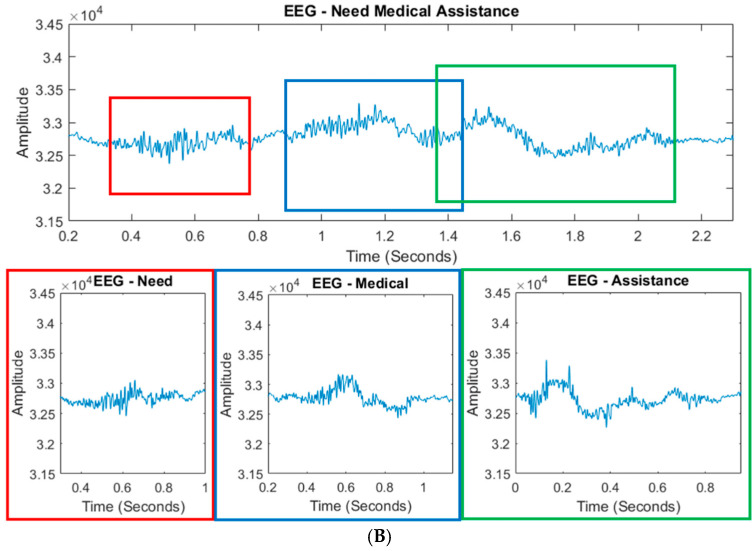
(**A**) EMG and (**B**) EEG recordings of the continuous sentence “Need Medical Assistance” from Corpus B, shown alongside individual word recordings from Corpus A for comparison.

Corpus B comprises 400 samples, with 100 recordings for each of the four sentences−”Need Medical Assistance”, “Land Here”, “Do Not Land Here”, and “Pick Us Up”. This dataset is used to retrain the SNN model to recognize words as they appear in continuous speech, accounting for inter-word signal distortions. Figure 4 displays both the continuous EMG and EEG signals for a sentence (top plots) and the corresponding individual word signals from Corpus A (bottom plots). Colored boxes highlight the approximate locations of individual words within the continuous sentence signals and reveal distortions caused by coarticulation and temporal overlap. These visualizations clearly demonstrate that simply concatenating isolated word signals cannot accurately reconstruct sentence-level signals. As a result, segmenting words from continuous input is highly challenging, and prior studies have often relied on manual pauses between words to facilitate segmentation—a method that reduces realism and practical applicability.

### 2.3. Few-Shot Learning: Siamese Neural Network (SNN)

Few-shot learning is a ML paradigm that enables accurate classification using only a small number of labeled examples, making it especially well-suited for SSR, where large-scale labeled datasets are difficult to obtain from human subjects. Among few-shot learning approaches, the SNN offers an effective framework by learning to compare input samples to a limited set of reference examples using a similarity-based metric.

In this study, we used an SNN to process EMG signals. The network consists of two identical convolutional subnetworks that share weights and biases. Rather than outputting class probabilities like conventional classifiers, the SNN computes a similarity score between input and reference samples in a shared feature space. During training, a contrastive loss function is used to minimize the distance between embeddings of samples from the same class and maximize the distance between those from different classes. Reference samples, which are preselected signals representing each class, act as anchors that structure the feature space into well-separated clusters.

**Figure 5:**
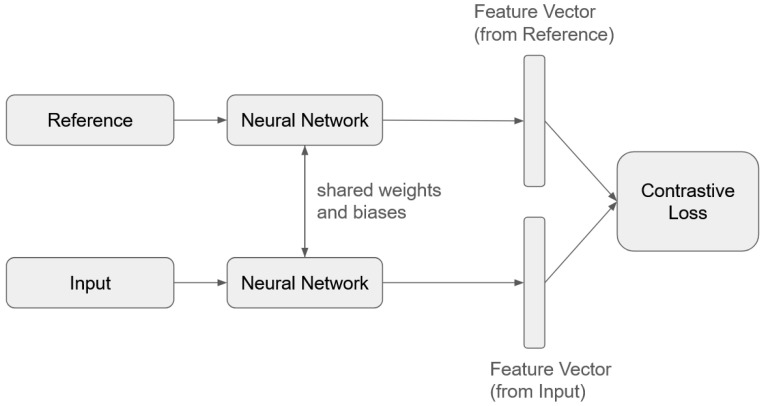
SNN Architecture.

At inference time, the model classifies new inputs by comparing them to the stored reference samples and selecting the class with the smallest distance. This architecture allows the SNN to generalize effectively from limited data, making it particularly advantageous for SSR applications. A simplified schematic of the SNN architecture is shown in Figure 5.

Input vectors are labeled as 1 or 0 based on their class relative to a reference sample: a label of 1 indicates matching classes (e.g., signals of the same word), while 0 indicates different classes. During training, pairs of input and reference signals are passed through the network. When the label is 1, the contrastive loss function minimizes the distance between their feature representations; when the label is 0, the loss function increases the distance. This iterative process encourages embeddings of similar samples to cluster closely in the feature space, while pushing dissimilar samples farther apart. As a result, the network forms distinct clusters, each corresponding to a unique class.

Once training is complete, reference embeddings for each class are stored for future comparison. During inference, a new input signal is passed through the network, and its distance to each reference embedding is computed. The input is then classified based on proximity in the feature space: smaller distances indicate greater similarity. The class associated with the reference vector yielding the minimum distance is selected as the prediction.

SNNs are particularly well-suited for scenarios with limited training data. As a few-shot learning approach, they require only a small number of labeled examples per class, and in some cases, a single high-quality reference signal is sufficient to represent a new class. Moreover, adding new classes does not require retraining the network, offering a flexibility that is uncommon in traditional classification models.

### 2.4. Augmentation

**Figure 6:**
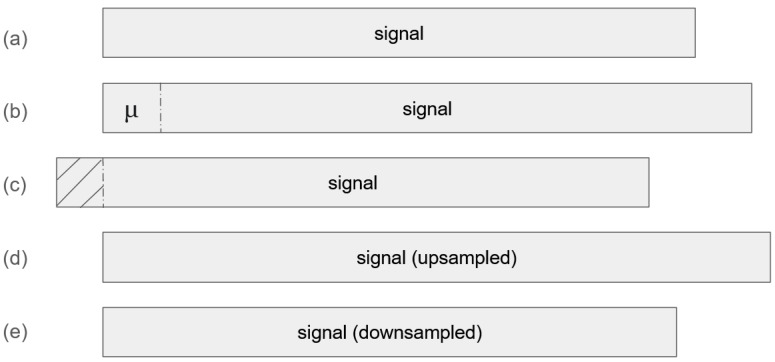
Augmentation of a sentence-level EMG/EEG signal: (**a**) original signal, (**b**) shifted signal with μ-padding, where μ is the average value of the signal, (**c**) cropped signal, (**d**) upsampled signal using interpolation, and (**e**) downsampled signal using interpolation.

Data augmentation is especially valuable when labeled training data is limited, as is often the case in SSR studies. In this work, we augmented the experimentally collected EMG and EEG signals through a combination of shifting, cropping, upsampling, and downsampling operations. The upsampling and downsampling processes were performed using interpolation techniques. A simplified overview of the augmentation methods is presented in Figure 6.

Beyond addressing data scarcity, augmentation in our study also serves a secondary purpose: it enables the model to better interpret partial word signals within continuous sentence recordings. By artificially shifting and modifying signal segments, the model gains robustness to temporal variations and boundary distortions. This augmentation strategy plays a critical role in the moving window-based retraining process, as discussed further in Section 2.5.2.

### 2.5. Moving Window-Based Word Segmentation and Classification

To enable word segmentation and classification from continuous sentence-level signals, we propose a moving window-based approach. This mechanism eliminates the need for manual pauses between words during data collection, thereby enhancing the practicality of our SSR system for real-world applications. The process involves three main steps: (1) pretraining on isolated words, (2) retraining using the moving window on sentence data, and (3) word classification from sentence-level signals.

#### 2.5.1. Pretraining Reference Model on Words

Before processing sentence signals, the model must first learn to recognize individual words. To this end, we pretrained an SNN model on the experimental and augmented dataset of Corpus A.

We adopted a one-dimensional convolutional neural network (1D-CNN) as the backbone of the SNN, shown in Figure 5, for efficient signal processing. Convolution was applied with a kernel size of 7 and padding of 3 to extract feature vectors from both reference and input signals. Cosine similarity was used as the distance metric in the contrastive loss, allowing the network to compare the input to reference vectors in a shared feature space.

For each word class, we selected five representative samples from the experimental data in Corpus A to construct the reference set (“shots”) used in the few-shot learning framework. The vocabulary includes 11 classes—10 target words and one noise class—resulting in a total of 55 reference signals. The remaining samples from Corpus A, along with their augmented variants, were used for training. During this phase, the model learned to adjust the shared parameters of the 1D-CNN to bring embeddings of the same class closer together while pushing those of different classes further apart in the feature space. This pretraining stage served to establish a foundational model capable of recognizing isolated words, thereby enabling subsequent segmentation and classification of words within continuous sentence-level signals.

#### 2.5.2. Moving Window-Based Retraining Model on Sentences

To enable word segmentation from continuous sentence signals without relying on manual annotations, we employed a moving window technique during the retraining phase. The pretrained SNN was further trained using both the experimental and augmented data from Corpus B. In this stage, input data consisted of cropped signal segments generated by sliding a fixed-size window across the sentence recordings. The original 55 reference signals described in Section 2.5.1 were retained for comparison during classification. For retraining, approximately 70% of the Corpus B data was used, with the remaining 30% reserved for testing.

**Figure 7:**
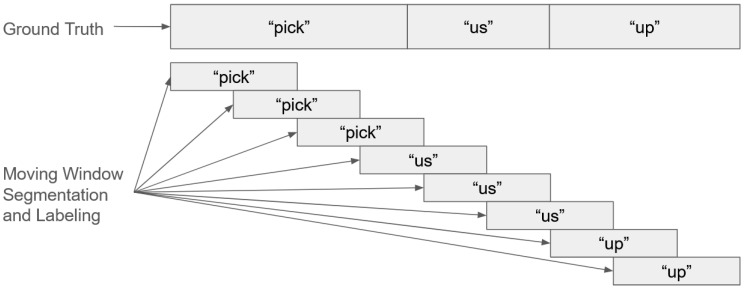
Moving window-based labeling for retraining.

The moving window was set to a size of 700 px with a stride of 380 px, selected via hyperparameter tuning. The window was made large enough to capture sufficient signal characteristics for word identification while remaining small enough to detect short words. Overlapping windows helped preserve transitional information between words. Ground truth labels for cropped segments were assigned based on their position within the sentence, under the assumption of evenly spaced words. While this assumption may not always hold, the augmented data enabled the model to adjust for misalignment during training. A schematic of the moving window-based retraining process is shown in Figure 7.

#### 2.5.3. Word Classification

After training, the final model was evaluated on the remaining samples in Corpus B. During inference, the moving window slides over the sentence signal, extracting snippets that are compared to each class’s reference signals. Each snippet is evaluated against all 5 reference signals per word class, and the cosine distances are averaged.

The candidate words are then ranked based on their average distances, and the word with the smallest average distance is selected as the final classification result. This process is repeated across the entire sentence to produce a sequence of predicted words, forming the basis for sentence reconstruction and further post-processing.

### 2.6. Corrections by Language Models

LMs can be effectively integrated into SSR systems to improve classification results, following the well-established practice in ASR. In ASR, it is common to post-process preliminary transcriptions with LMs to enhance overall accuracy, fluency, and syntactic consistency. Motivated by this success, we adopt a similar strategy in SSR to correct misclassified words and produce more coherent and grammatically valid sentences.

In particular, we employ an *n*-gram LM to guide the correction process by leveraging the statistical properties of word sequences. An *n*-gram refers to a contiguous sequence of *n* words or characters. These models are typically derived from large text corpora by computing the frequency of occurrence for each possible *n*-gram [28]. The resulting probabilities reflect common usage patterns in the language. To handle rare or previously unseen *n*-grams and avoid the zero-probability issue, smoothing techniques are applied.

During inference, the trained SNN model generates a ranked list of word candidates for each segment of the sentence-level EMG/EEG signal, along with associated classification probabilities. The *n*-gram model is then applied to evaluate the syntactic plausibility of these candidate sequences. Implausible or statistically rare *n*-grams are filtered out, and the remaining candidates are re-ranked based on their *n*-gram likelihood. A cutoff threshold *K* is applied to select the top *K* most probable sentence candidates.

**Figure 8:**
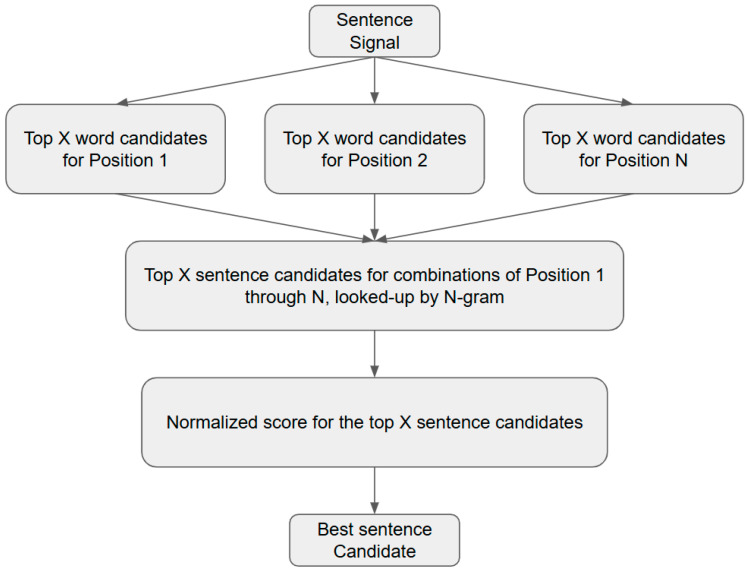
Flowchart of sentence correction using a tri-gram LM.

For each of these *K* candidates, a confidence score is calculated by combining the *n*-gram probability with the original classification probabilities from the SNN model. These scores are normalized based on the number of words in each candidate sequence. The candidate with the highest combined confidence score is selected as the final corrected transcription. Figure 8 illustrates this correction process using a tri-gram model as an example.

By incorporating an *n*-gram LM into the SSR pipeline, we are able to exploit statistical regularities in the English language, thus improving the robustness and accuracy of sentence-level predictions.

## 3. Results

To evaluate the effectiveness of our SSR framework, three models were developed and tested under a range of experimental conditions. The first model, Model A, was trained exclusively on isolated word signals from Corpus A (150 samples for each word totalling 1500 samples) using an SNN with a one-dimensional convolutional architecture. The dataset was split into 70% for training and 30% for testing. The second model, Model B, was initialized with Model A’s weights and retrained on sentence-level data from Corpus B (100 samples for each sentence totaling 400 samples) using a moving window mechanism. This allows the model to adapt to the signal distortions and coarticulations present in continuous speech. Again, 70% of the data was used for retraining and 30% for testing. The third model, Model C, extended Model B by incorporating a trigram LM as a post-processing step to correct structurally implausible or misordered word predictions.

Performance was evaluated across three recognition targets: isolated words, words within sentences, and complete sentence recognition. Additionally, we compared three sensor configurations: EMG only, EEG only, and sensor fusion using both EMG and EEG. Classification accuracies for all combinations are presented in Table 2.

When tested on isolated word signals, Model A achieved high classification accuracy across all sensor types. Average word recognition accuracy was 90.56%, with sensor fusion yielding the highest performance at 98.67%, followed by EMG at 90.33%, and EEG at 82.67%. These results indicate that the SNN effectively learned discriminative features for word classification in clean, non-overlapping conditions.

However, Model A’s performance declined sharply when applied to words embedded in sentence-level signals. The average accuracy dropped to just 24.20%, with EMG, EEG, and fusion accuracies falling to 29.15%, 17.10%, and 26.36%, respectively. This drop is expected, as the model had not been trained on temporally distorted or blended word signals that are typical in continuous speech. These results highlight the limitations of isolated word training for real-world SSR applications.

Retraining the model using the moving window approach substantially improved performance. Model B achieved an average word-in-sentence accuracy of 85.46%, with sensor fusion again performing best (91.24%), followed by EMG (85.07%) and EEG (80.06%). The improvement demonstrates that exposing the model to distorted, context-rich signals allow it to generalize better to real-world conditions. Despite this improvement at the word level, sentence recognition remained a challenge. For sentence-level evaluation, we removed repeated words from predicted sequences (e.g., “Pick us us up” was reduced to “Pick us up”) to align predictions with the reference labels. Model B achieved sentence accuracies of 73.41% for EMG, 52.16% for EEG, and 93.30% for fusion signals. The slightly higher performance in the fusion case is likely due to the additional signal information compensating for word-level inconsistencies.

To further enhance sentence recognition, we applied a locally trained trigram LM to Model B’s output, resulting in Model C. The LM filtered and re-ranked candidate word sequences based on their syntactic likelihood, correcting sequences that were semantically or grammatically implausible. This post-processing step significantly improved sentence recognition accuracy across all sensor types. EMG accuracy increased from 73.41% to 93.33%, EEG rose from 52.16% to 87.83%, and sensor fusion improved from 93.30% to 95.25%, bringing the overall average to 92.14%.

The findings confirm that training with sentence-level data is crucial for accurate recognition in continuous speech settings. They also demonstrate the value of language modeling in correcting residual prediction errors and improving syntactic coherence. Notably, sensor fusion consistently outperformed unimodal configurations in nearly all scenarios, underscoring its importance in creating robust SSR systems. Together, these results support a multi-stage approach to SSR, in which few-shot learning, temporal modeling, and language-based correction work in concert to enable high-accuracy sentence prediction from biosignals.

## 4. Discussion

This study presents a complete SSR system that combines few-shot learning, sensor fusion, and LM to improve sentence-level recognition from EMG and EEG signals. The approach was designed to handle challenges such as signal distortion in continuous speech and the limited availability of labeled training data. The results show that combining signal processing techniques with language-based correction leads to better performance and more reliable predictions.

A key strength of this work is the use of a wearable, low-channel sensor configuration. Unlike previous SSR studies that rely on high-density or multi-channel EMG and EEG systems, we implemented a wireless, single-channel EMG and single-channel EEG setup. This minimal sensor configuration makes the system lightweight, portable, and more practical for real-world use, especially in field-deployable or mobile environments. Despite the reduced hardware complexity, our framework achieved high recognition accuracy—comparable to or exceeding that of more complex systems—by leveraging algorithmic strategies such as sensor fusion and LM-based correction.

A consistent finding across all models (A, B, and C) is that EMG outperformed EEG in both word-level and sentence-level recognition. This result is expected and aligns with the functional properties of the two modalities. EMG directly captures muscle activity from the tongue and jaw—structures that have a one-to-one correspondence with articulatory movements during speech production. These signals are temporally precise and provide rich, discriminative features for distinguishing phonemes and words. In contrast, EEG reflects broader cortical activity related to speech planning and motor control, typically measured with more signal attenuation and lower spatial resolution. This makes it more challenging to isolate speech-specific neural patterns, especially in a single-channel, wearable configuration.

Another important observation is that training a model only on individual word signals is not enough for recognizing words within full sentences. In real speech, words are not clearly separated—they often blend together due to natural transitions between sounds. These transitions change the EMG and EEG signals, especially at the beginning and end of each word. Models that are not trained on these types of signals struggle to recognize words correctly in continuous sentences. By retraining the model using a moving window to simulate real sentence conditions, we enabled it to learn how words appear when spoken naturally. This made the model more effective for practical SSR tasks.

Sensor fusion—combining EMG and EEG data—further improved the model’s ability to understand silent speech. Each signal type provides different information: EMG captures muscle activity, while EEG records brain signals related to speech planning. By using both together, the model could make more informed decisions, especially in noisy or uncertain cases. This combination made the system more reliable and less sensitive to the weaknesses of any one sensor type.

LMs also played a crucial role in enhancing sentence-level recognition performance within our SSR system. While the SNN produced word predictions based on biosignal features, these raw outputs frequently lacked grammatical coherence or semantic plausibility. To address this, we integrated a trigram LM trained on the same set of military command phrases used in the experiments. This LM served as a post-processing filter, evaluating candidate sequences for linguistic consistency and correcting many syntactic or lexical errors. As demonstrated in Table 3, the LM successfully removed extraneous or misplaced words in several instances. For example, the misclassified sequence “Need Medical Assistance Land” was corrected to the intended phrase “Need Medical Assistance”. These corrections significantly improved overall sentence-level accuracy by aligning the final outputs with valid, task-relevant language patterns.

These findings reinforce the conclusion that building a high-performing SSR system requires more than effective signal classification. Accurate sentence recognition depends on a comprehensive pipeline that includes (1) models trained on realistic, continuous speech-like signals, (2) sensor fusion to capture complementary information across modalities, and (3) LMs that refine raw predictions based on prior linguistic knowledge. The resulting system is capable of accurate, silent communication from limited training data, making it well suited for deployment in sensitive or constrained environments—such as military operations, assistive communication for individuals with speech impairments, or secure communication where acoustic output is impractical or undesirable.

## 5. Conclusions

In this study, we presented a silent speech recognition (SSR) framework that combines a moving window-based few-shot learning model, sensor fusion of EMG and EEG signals, and post-processing with a statistical language model (LM). This method aims to enable accurate sentence recognition from continuous, non-vocalized biosignals without requiring manual word segmentation.

To demonstrate the feasibility of the proposed framework, we tested sentence-level recognition accuracy on four military commands using simultaneous EMG and EEG signals collected from a wearable sensor system. It also demonstrates that sentence-level SSR systems can be developed effectively using small datasets and a limited number of reference samples, enabled by a few-shot learning approach, which is especially valuable in real-world applications, where large-scale biosignal data collection is often impractical. The feasibility test achieved a sentence-level accuracy of 95.25% using a single-channel, wireless EMG/EEG configuration. The few-shot learning model was designed to perform effectively with limited training data, and the moving window approach enabled the model to learn word patterns within continuous speech signals without requiring artificial pauses or manual segmentation. Sensor fusion improved recognition by combining complementary information from both muscular and neural signals, while the trigram language model (LM) further enhanced accuracy by correcting syntactic and lexical errors in the predicted word sequences.

Importantly, this work shows the feasibility of achieving high-performance SSR without complex hardware. By combining algorithmic innovations with a minimalist sensor setup, the system can achieve both high accuracy and practical usability for real-world deployment.

Future work will focus on improving the generalizability and scalability of the system. First, we plan to expand data collection to include a wider range of sentences and vocabulary, as well as a diverse participant pool. This will enable a comprehensive evaluation of the model’s robustness across different users. Second, we plan to assess the system’s performance under real-world conditions by studying the impact of motion artifacts and environmental noise, with the goal of enhancing its reliability and resilience in practical settings. Third, we plan to implement more advanced neural architectures, such as Transformer-based models, which may offer improved feature extraction and sequence modeling capabilities. Fourth, we aim to shift from word-level classification to phoneme-level prediction. With only 44 phonemes in the English language, this approach could enable broader generalization across vocabularies, making the system adaptable to any phrase without retraining on specific word sets.

In summary, the proposed framework demonstrates that accurate, wearable, and sentence-level SSR is feasible using low-channel biosignal inputs. The integration of few-shot learning, sensor fusion, and LM forms a scalable foundation for silent, efficient, and context-aware communication systems in military, medical, and secure environments.

## Figures and Tables

**Table 1:** The specifications of the wearable and wireless EEG/EMG sensor system.

Specification	Description	Specification
Power Source	Rechargeable Battery	Capacity: 80 mA
Data Transmission	BLE	Speed: 1 Mbps in 2 m
EMG Electrodes	Disposable Ag/AgCl Standard	Size: 20 × 20 mm
Front-End Circuit	Intan Tech Chip (RHD2216)	10 mV, 16 bit, 16 ch
Onboard CPU	ARM Cortex M4	Sampling Rate: 4096 Hz/ch
Wireless Circuit	NRF 52X	Speed: 2.4 GHz, BLE

**Table 2:** Word and sentence recognition accuracies.

Accuracy	Word Accuracy	Word Accuracy	Word Accuracy	Sentence Accuracy	Sentence Accuracy
Model	Model A	Model A	Model B	Model B	Model C
Target	(a) Individual Words	(b) Words in Sentence	(b) Words in Sentence	(c) Sentences	(c) Sentences
EMG	90.33%	29.15%	85.07%	73.41%	93.33%
EEG	82.67%	17.10%	80.06%	52.16%	87.83%
Fusion	98.67%	26.36%	91.24%	93.30%	95.25%
Average	90.56%	24.20%	85.46%	72.96%	92.14%

**Table 3:** Examples of LM Corrections.

Ground Truth	Wrong Prediction by SNN	LM Correction
“Need Medical Assistance”	Need Medical Assistance Land	Need Medical Assistance
“Do Not Land Here”	Do Not Land Need Here	Do Not Land Here
“Land Here”	Pick Land Here	Land Here
“Pick Us Up”	Pick Us Up Here	Pick Us Up

## Data Availability

The original data presented in the study are openly available in Google Drive at https://drive.google.com/drive/folders/1GOaop2wyi8MXKqMXnaxYamodVEMCnY_v?usp=sharing (accessed on 29 September 2025).
